# Deconvolution of Complex DNA Repair (DECODR): Establishing a Novel Deconvolution Algorithm for Comprehensive Analysis of CRISPR-Edited Sanger Sequencing Data

**DOI:** 10.1089/crispr.2020.0022

**Published:** 2021-02-19

**Authors:** Kevin Bloh, Rohan Kanchana, Pawel Bialk, Kelly Banas, Zugui Zhang, Byung-Chun Yoo, Eric B. Kmiec

**Affiliations:** ^1^ Gene Editing Institute, Helen F. Graham Cancer Center and Research Institute, ChristianaCare, Newark, Delaware, USA.; ^2^ Department of Medical and Molecular Sciences, College of Health Sciences University of Delaware, Newark, Delaware, USA.; ^3^ Value Institute, ChristianaCare, Newark, Delaware, USA.

## Abstract

During CRISPR-directed gene editing, multiple gene repair mechanisms interact to produce a wide and largely unpredictable variety of sequence changes across an edited population of cells. Shortcomings inherent to previously available proposal-based insertion and deletion (indel) analysis software necessitated the development of a more comprehensive tool that could detect a larger range and variety of indels while maintaining the ease of use of tools currently available. To that end, we developed *De*convolution of *Co*mplex *D*NA *R*epair (DECODR). DECODR can detect indels formed from single or multi-guide CRISPR experiments without a limit on indel size. The software is accurate in determining the identities and positions of inserted and deleted bases in DNA extracts from both clonally expanded and bulk cell populations. The accurate identification and output of any potential indel allows for DECODR analysis to be executed in experiments utilizing potentially any configuration of donor DNA sequences, CRISPR-Cas, and endogenous DNA repair pathways.

## Introduction

CRISPR-directed gene editing has established itself as a technically feasible platform for the genetic modification of eukaryotic cells. The CRISPR-Cas system enables a variety of targeted edits to be introduced into specific genes or to non-coding regions in the genome.^1,2^ The initialization of the gene-editing process includes the generation of a double-strand break (DSB) at one or more predefined sites. DSBs trigger a DNA damage response in the cell, activating pathways such as non-homologous end joining (NHEJ), microhomology-mediated end joining (MMEJ)/single-stranded annealing, or, in the presence of a donor DNA template, homology-directed repair (HDR).^3–6^ These pathways do not operate independently, and the interplay among them can lead to a complex mixture of unedited DNA and edited DNA ends harboring various insertions and deletions (indels) surrounding the cleavage site.^7–9^ It is the unpredictability and diversity of edited outcomes within a population of cells that have raised caution as CRISPR-directed gene-editing programs advance toward clinical application. Thus, there is a need to develop and refine analytical tools that can provide an accurate detailed global view of the products of such genetic modification, particularly in human cells.

Targeted deep sequencing is considered by many to be the most comprehensive method to obtain an accurate description of all relevant indels present in a genetically modified cell population.^10–13^ For many investigators, however, target deep sequencing is costly, and the turnaround time prevents simple high-throughput implementation. Research groups also make use of Sanger dideoxynucleotide sequencing to generate analyzable indel data. While this technique can provide important information through allelic analysis when one to three alleles are present in a clonal population, research groups often seek to identify the array of indels in a targeted bulk population of cells, which could contain thousands of indel-diverse sequences.

Several analytical tools, including TIDE (Tracking of Insertions and DEletions; Desktop Genetics), ICE (Inference of CRISPR Edits; Synthego), and CRISP-ID (KU Leuven), have also been developed. All three programs deconvolute Sanger sequencing data so that they can be read as a series of sub-sequences, each of which corresponds to an indel within an individual allele. Upstream of the cleavage site in an edited bulk DNA sequence, the *base calls* at each nucleotide position are synchronous; although multiple ddNTP-tailed sequences are being analyzed, their alignment is identical. Starting at the onset of indels, however, these individual ddNTP-tailed DNA fragments begin to appear as shifted sequences, created through the myriad indel-containing alleles present in the whole population. When this mixed population of indels (and wild type) is present, the sequence degenerates immediately, with multiple peaks appearing at nearly every position in the sequence's chromatogram. To resolve the sequence hybrids, TIDE, ICE, and CRISP-ID make use of a control Sanger output file and an experimental Sanger output file containing the data of the bulk edited sequence. The programs establish an “alignment window” upstream to the onset of indel formation, where the initial reference frame is created to orient any sequence shift found downstream. Afterwards, the programs create a “decomposition/inference window,” which corresponds to a stretch of bases located a certain distance downstream of indel onset, within the decomposed sequence.^14–16^

TIDE's output consists of a bar plot consisting of net indel magnitudes detected within the inference window, with individual indels listed as significant or not via a two-tailed *t*-test of the variance–covariance matrix of the standard errors. ICE, conversely, returns a list of full sequences determined as present within the inference window. Detectable indels fall within a ±50 bp range for TIDE and −30/+14 bp for ICE, though insertions are labeled with ambiguity code N. ICE also allows for submission of up to three gRNA sequences, though more than one gRNA further shrinks the indel range to −10 to +3 bp.^14,15,17,18^

Since their introduction, both TIDE and ICE have also evolved a usage for estimating the frequency of HDR. TIDER (Tracking of Indels, DEletions and Recombination events; Desktop Genetics) allows for knock-in percentages to be estimated with the addition of a template sequence, a Sanger sequencing file containing a known DNA change.^17^ ICE, with its “ICE v2” update, carries a similar functionality, although it allows for the input of the template sequence to be input in text format, rather than requiring an .ab1 Sanger sequencing file.

While TIDE, ICE, and CRISP-ID enable important and useful indel analyses, common limitations impede detailed or difficult indel analyses. The methods used by all three programs utilize edit proposals that are determined primarily by the shift in distance between analysis and inference window, moving the resulting sequence upstream in the event of a deletion or downstream in the event of an insertion. If more than a single base is added, as is very common in CRISPR-editing reactions, TIDE and ICE only have the capability of determining the amount, not identity, of these inserted bases. Another limitation includes a restriction on the length of indels capable of being analyzed; both programs are restricted to ≤50 bases around each cleavage site. The stochastic nature of DNA repair after CRISPR cleavage makes deletions outside this range common in clonal analysis, especially when multiple different gRNAs are being utilized. ICE only allows for the analysis beyond this range if two or three gRNAs are used, which further shrinks the edit window around each cut site. Any indels that fall outside of these limited ranges cannot be aligned by these algorithms. CRISP-ID is also limited in accuracy to tri-allelic clonal populations, precluding its use in determining any bulk editing efficiencies.^18^ This can prevent accurate determination of the contributions of real sequences and lead to mistaken identification of false-positives.

The shortcomings inherent to previously available proposal-based indel analysis tools necessitated the development of a more comprehensive software tool that could detect a larger range of indels while maintaining the ease of use of the currently available tools. To that end, we developed *De*convolution of *Co*mplex *D*NA *R*epair (DECODR). DECODR can detect indels formed from single or multi-guide CRISPR experiments without a limit on indel size. DECODR utilizes a unique proposal generation and determination algorithm that allows accurate uncovering of indel identities, including those of inserted bases. This greater accuracy of genetic content determination allows users to be better informed about the exact genetic changes their CRISPR edits are creating.

## Methods

### Sanger sequencing and manual sequence deconvolution

For each live-cell sample, genomic DNA was isolated from each sample using the DNeasy Blood and Tissue Kit (Qiagen). The regions surrounding each target site were amplified according to the parameters shown in Supplementary Table S1. The polymerase chain reaction (PCR) reaction was purified using the QIAquick PCR Purification Kit (Qiagen) and sequenced using a SeqStudio Genetic Analyzer (Applied Biosystems), providing .ab1 files for analysis and testing.

For manual sequence deconvolution, an alignment window was established by listing the DNA sequence prior to the decomposition point and determining the position on the wild-type or reference Sanger file where this sequence was located.^19^ Then, at each position after the decomposition point of the sequence, each subpeak that could be visualized at each position was recorded, creating an array containing all relevant sequence information. Using positions of low complexity where only one peak was visualized, manual edit proposals were generated based on net indel size (sequence shifted up- or downstream X bases), after which the edit proposals were eliminated or confirmed by cross-checking each position with the basecall identity that would appear at that position in the shifted edit proposal. Using this method, two-allele convoluted sequences could be deconvoluted with minimal difficulty, and three-allele convoluted sequences with a higher rate of error. Four-allele and bulk sequences could not be deconvoluted this way, and manual deconvolution also did not allow for the determination of sequence contribution percentages.

### Next-generation sequencing

For next-generation sequencing (NGS), genomic DNA was isolated from each sample using the DNeasy Blood and Tissue Kit (Qiagen). The regions surrounding each target site were amplified using primers that contained partial Illumina adapter sequences (Illumina TrueSeq Paired-End adapters v3). PCR products of 497 bp were isolated using the QIAquick PCR Purification Kit (Qiagen), and gel electrophoresis was performed to verify no nonspecific peaks were generated. The sample was sent for NGS at Genewiz (Genewiz, Inc., South Plainfield, NJ) using their “Amplicon-EZ” service. Raw fastq files were returned. Sequence quality was determined using FastQC v0.11.5.^20^ Data were then analyzed using CRIS.py v2.^21^

### Cell culture and transfection

All cell lines described were maintained at 37°C and 5% CO_2_. K562 cells were cultured in Iscove's Modified Dulbecco's Medium (ATCC), supplemented with 10% fetal bovine serum (FBS; ATCC) and 1% penicillin/streptomycin antibiotics. Hel92.1.7 were cultured in ATCC-formulated RPMI-1640 (RPMI-1640 containing 2 mM l-glutamine, 10 mM HEPES, 1 mM sodium pyruvate, 4,500 mg/L glucose, and 1,500 mg/L sodium bicarbonate), supplemented with 10% FBS (ATCC) and 1% penicillin/streptomycin. CL9 cells were cultured in Dulbecco's modified Eagle's medium (ATCC), supplemented with 10% FBS (ATCC). NCI-H1703 cells were cultured in ATCC-formulated RPMI-1640 (RPMI-1640 modified to contain 2 mM l-glutamine, 10 mM HEPES, 1 mM sodium pyruvate, 4,500 mg/L glucose, and 1,500 mg/L sodium bicarbonate), supplemented with 10% FBS (ATCC). LNCAP cells were cultured in Improved Minimum Essential Medium, supplemented with 5% FBS (ATCC).

For plasmid transfections, K562 and Hel92.1.7 cells were transfected and cloned as described previously.^22^ For all transfections of CL9, H1703, and LNCAP cells, a Lonza Nucleofector 4D (Lonza, Inc., Basel, Switzerland) was used. For transfection, cells were seeded 48 h prior and allowed to reach 60–80% confluency. On the day of transfection, cells were harvested by trypsinization and washed twice with 1 × phosphate-buffered saline (–/–). Cells were re-suspended at 3 × 10^5^ cells/20 μL in appropriate solution (CL9: P3, H1703: SF, LNCAP: SF)/supplement solution, and 20 pmol RNP complex was added for each sample. Lonza programs CA-137, CM-130, and EN-120 were used for CL9, H1703, and LNCAP cells, respectively, and after 15 min of rest, cells were transferred to a six-well plate for further growth.

### DECODR software

The DECODR deconvolution algorithm was written in Python v3.6.8. All modules and libraries implemented within the software are either open source or custom written. A list of utilized software and libraries are included in the Supplementary Data (Supplementary Table S2). Input files consist of one control file per analysis (plain text, FASTA, or .ab1) and any number of experimental files (FASTA or .ab1), along with up to two CRISPR guide sequences (plain text), an optional homology donor DNA sequence (plain text), and a nuclease (Cas9 or Cas12a). If text-based files are provided, they are interpreted as chromatograms with identical peak heights for each position.

The front-end user interface was written as a single-page application with the React.js library, and the back end was written with the Django web framework using Celery and Redis to handle long-running analyses.

**FIG. 1.**
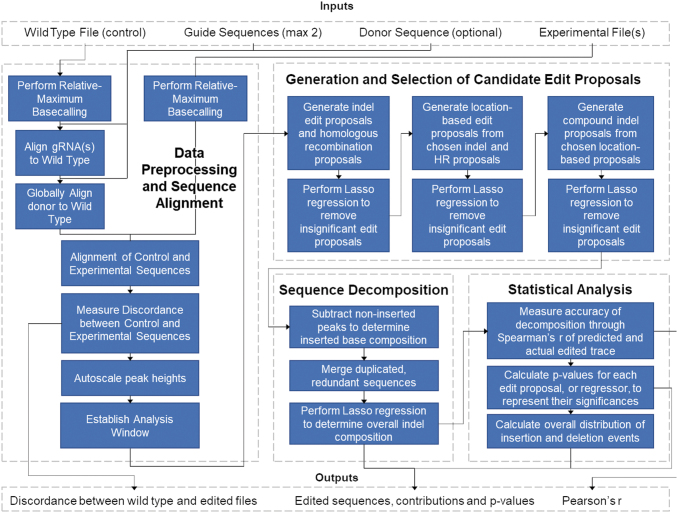
DECODR algorithm flow chart. Input files consist of one control file per analysis (plain text, FASTA, or .ab1) and any number of experimental files (FASTA or .ab1), along with up to two CRISPR guide sequences (plain text) and an optional homology donor DNA (plain text). The algorithm first checks data quality and performs trimming and peak equalization for both the control and experimental files. Control and experimental files are aligned in order to establish an inference window for each input experimental file. Insertion and deletion (indel) classes are determined based on the presence of broad classes of indel ranges in order to minimize the amount of individual edit proposals. Overall indel composition is determined via Least Absolute Shrinkage and Selection Operator regression, after which inserted base identities are determined. The program outputs a visual readout consisting of a summary of indels seen in the entire test set (if multiple experimental files were input) along with specific summaries for each experimental file.

A flow chart of the DECODR algorithm can be seen in Figure 1. First, DECODR determines all peak values (relative local maxima in signal intensity) and their locations across the sequencing trace for each of the four nucleoside channels. Within a base-pair location (BPL), base calls and their intensities are determined from the local maximum values that lie closest to the BPL. This process is repeated for each BPL in the sequencing trace. DECODR then adjusts these data to counteract the gradual decrease in average signal intensity post decomposition: for each successive group of 10 adjacent BPLs, DECODR scales all contained base intensities by a constant rate *k* such that the arithmetic mean—of the 10 total base intensities at each BPL—is equal to an arbitrary predefined constant of 10,000. This adjustment sets a constant average height for these base intensities while also maintaining the relative differences in base intensities of adjacent BPLs.

Next, the guide sequence(s) are aligned to the control to find the location of the cut site(s) in the experimental trace. If a donor sequence is provided, it is aligned to the control sequence using the Needleman–Wunsch global alignment algorithm, implemented with the pairwise2 package in Biopython (v1.76).^23^ The control and experimental traces are aligned using the region of exact sequence homology that occurs before editing begins in the experimental trace. Subsequent determination of indels and other edits are based on deviations from this “control” alignment.

DECODR then considers the start of CRISPR editing as the BPL in which frequent deviations from the “control” alignment in the experimental trace begin. Strictly, the “inference data,” or base intensity data, contained in the region of 120 BPLs (potentially <120 BPLs for lower-quality traces) after the start of CRISPR editing are used to determine the editing events.

To determine these events, we expand upon previous methods that treat Sanger sequencing deconvolution via linear regression modeling. Briefly, the inference data are considered a dependent outcome linearly regressing on the unknown actual sequences of the CRISPR editing edits. To determine these unknown sequences, up to thousands of potential sequences are generated from potential CRISPR edits on the control sequence, represented as a regressor matrix, and run through a linear regression in which the response variable is the inference data.^14,15^

Given that we have a larger number of potential sequences, we use Least Absolute Shrinkage and Selection Operator (Lasso), with the sum of the absolute values of weights as the following penalty to reduce the data set to only the most important features that would impact the “target variable,” that is, the inference data:

$$min\left(\sum_{i=1}^{n}\left|y_{i}-w_{i}x_{i}\right|+\lambda \sum_{i=1}^{n}\left|w_{i}\right|\right)$$

Specifically, we use a non-negative Lasso regression (*λ* = 1.0) implemented with scikit-learn (v0.22.1) to determine the coefficients for each regressor or potential sequence.^24^ Subsequent scaling of these coefficients such that their sum is 100 provides the relative linear contribution for each potential sequence as a percentage. *p*-Values are calculated with a previously used two-tailed *t*-test.^14^ Any potential sequences with contribution <0.5% or *p*-value >0.05 are not considered in the inference data, and so they are removed. Pearson's correlation coefficient *r* is determined using the *pearsonr* function in SciPy (v1.2.0) to measure the goodness of fit.^25^

However, the number of potential sequences to run through this Lasso regression and selection procedure grows combinatorically large in accounting for an expanded search space of CRISPR edits comprised of compound indels (combined insertion and deletion events), multi-guide events, partial HDR events, and larger indel events. Previous approaches implicitly reduce the complexity of analysis by discounting these broader categories of edits.

To resolve this, instead of attempting to determine the CRISPR editing events with one linear regression, a combination of automated variable selection and multiple linear regressions together reduce the search space.^14,15^ This is enabled by the observation that potential sequences can be grouped by their net indel—the overall shift of the DNA subsequence downstream of the indel. The potential sequences within a given net indel group share exact sequence homology downstream of the cut site, so they are of highly collinearity. To handle this collinearity, DECODR runs an initial Lasso regression wherein only one potential sequence from each net indel group is used. Any potential sequences subsequently removed from consideration due to low contribution or high *p*-values (>0.05) also, due to mutual collinearity, remove from consideration the other potential sequences within its net indel group.

This reduces what is frequently more than 350 net indel groups to at most 10–15 net indel groups for one experimental trace. Within this reduced set of groups, all the potential sequences can be tested now, accounting for potential edit location by including all upstream or downstream shifts of each edit that still touch the cut site, potential compound indels by allowing up to four combined sets of one insertion and one deletion event for each edit, and potential partial HDR events. With this expanded set, another Lasso regression and selection step is run.

To determine the base identities of unknown insertions (non-HDR), we expand upon previous methods to determine the identity of +1 insertions.^14^ For an unknown inserted base, DECODR subtracts the other confirmed sequences' base intensities from the inference data at the given base's BPL. The resultant distribution between all four base identities is assigned to the inserted base, and this process is run for all inserted bases. However, in the case of multiple inserted bases within a BPL, a combined distribution of both base's identities is provided.

A final Lasso regression and selection step are run with these updated potential sequences, providing the contributions and *p*-values of each predicted sequence, predicted identities of inserted bases, and *r*^2^ value to measure goodness of fit. The program then outputs a readout containing the analyses of each submitted experimental file—the predicted sequences (and their contributions and *p*-values), a bar graph showing the contributions of each indel, and a live-updating stacked bar chart showing the base identity distribution for each inserted base—alongside an option to export the results to spreadsheet format.

The software is available at https://decodr.org/analyze. Requests for local access options or source code inquiries can be made to GeneEditingInstitute@ChristianaCare.org.

## Results

### DECODR can deconvolute clonal cellular targeted sequences

**FIG. 2.**
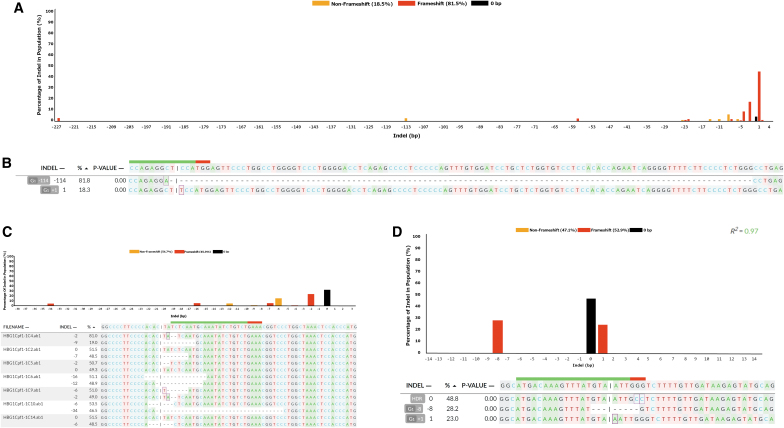
DECODR is efficient at analyzing poly-allelic DNA sequencing samples from clonally expanded cells. **(A)** Accurate determination of indel contributions were determined in a batch run of 30 biallelic clonal expansions targeted with a single Cas9 guide RNA targeting the GATA1 locus, as described here (Bloh KM, et al. 2017). The top pane displays the overall indel distribution of 30 clonal sequences. **(B)** An example of one of the largest recorded deletions that was verified by manual allelic analysis, listing a 115 bp deletion paired with a 1 bp insertion on one allele, and a separate 1 bp insertion on the second allele. **(C)** Clonal expansions of cells biallelic for the HBG1 locus were isolated and analyzed for editing efficiency using the DECODR software. Heterozygous and homozygous indel-containing sequences were accurately deconvoluted with indels ranging from 2 to 34 bp deletions. Sequences were confirmed by manual allelic analysis. **(D)** Clonal expansion resulting in tetra-allelic clones can be accurately deconvoluted with DECODR. TRM10C was targeted with a single Cas9 guide and a repair template oligo containing a 2 bp GG>CC change, resulting in two template-repaired alleles, one allele with a 8 bp deletion and one allele with a 1 bp insertion, confirmed by manual deconvolution. This tetra-allelic sample was successfully deconvoluted with the DECODR software, exhibiting accurate indel identities and an expected 1:2:1 contribution distribution.

In order to determine whether DECODR could accurately determine indel constituents and efficiencies in CRISPR-generated cellular targeting data, we utilized a data set published previously containing CRISPR-Cas9 cleavage products in K562 cells (Fig. 2A).^22^ This data set contained 30 biallelic clonal mixed sequences that were previously deconvoluted manually and contained a wide range of both insertions and deletions, and of “compound indels,” where bases are both inserted and deleted on the same allele.^19^ DECODR was capable of accurately determining the constituent sequences of each clonal population, with no limit on size of indel observed. Several clones isolated contained large deletions, including one clone containing a compound indel of −115 and +1 bp on one allele, and a +1 bp insertion on the other (Fig. 2B). Correct indel descriptions were also output when tested with another data set, containing clonal samples generated in Hel92.1.7 cells using both CRISPR-Cas9 and CRISPR-Cas12a (Fig. 2C).

Correct indel deconvolution could also be performed in tri-allelic and even tetra-allelic clonal samples within a third test data set that utilized Cas9 RNPs to edit CL9 cells (Fig. 2D). The three clones shown display a contribution ratio of approximately 2:1:1, indicating the presence of four alleles in each clonal population, with two alleles sample displaying an identical HDR-repaired indel pattern. Correct deconvolution of clonal populations across a variety of cell lines and CRISPR targets was performed to confirm viability (Supplementary Fig. S1A–C).

### DECODR can deconvolute bulk cellular targeted samples

After determining that DECODR had the capacity to analyze a wide range of isolated clonal populations containing four alleles or fewer, we next tested the capabilities of DECODR in analyzing “bulk” samples, containing amplicons generated from tens of thousands of un-isolated cells. Indel diversity seen in these samples is vastly higher than in clonal populations, making analyses of bulk samples much faster at determining initial CRISPR cleavage efficiency. To confirm DECODR's ability to deconvolute both single-cut and multi-cut populations, we analyzed bulk DNA samples using both DECODR and the established TIDE and ICE applications.

**FIG. 3.**
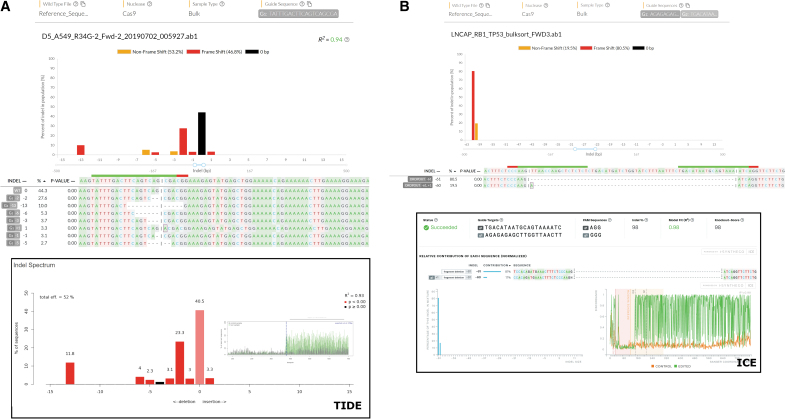
DECODR is efficient at analyzing poly-allelic DNA sequencing samples from bulk targeting experiments. **(A)** A single-guide experiment targeting the NRF2 locus in H1703 cells was performed using the two tools capable of statistical verification of individual edit proposals (TIDE and DECODR), with the DNA from the whole targeting reaction isolated and analyzed via DECODR (top panel). Analysis of the same file using TIDE is also displayed (bottom panel). **(B)** A dual-guide experiment without a homology donor was performed targeting the rb1 locus in LNCAP cells. Analysis was compared between the two tools capable of multi-guide analysis (ICE and DECODR). Indel analysis comparisons are displayed between DECODR (top panel) and ICE (bottom panel).

The single-cut bulk sequences were generated by targeting H1703 cells with a CRISPR-Cas9 RNP targeting NRF2 (Fig. 3A). The indel pattern displayed by DECODR showed an indel distribution weighted toward 2 and 13 bp deletions, with a lower level of other indels ranging from +1 to −6 bp. These indels were compared to the current software by analyzing the same sample with TIDE (Fig. 3A). The overall indel distribution is similar between both methods. Interestingly, the presence of a −4 bp indel is present in TIDE but absent in DECODR; this sequence contribution of <1% is below the variable selection threshold for this sample in DECODR. Likewise, in TIDE, the edit proposal is determined as nonsignificant.

Figure 3B shows a dual-cut bulk sequence of a gene-editing reaction targeting Rb1 in LNCAP cells with two gRNAs. The only significant products output by both DECODR and ICE were a full excision of the sequence between the two guides, resulting in a 61 bp deletion. Both ICE and DECODR also determine the presence of a sequence within the bulk of a −60 bp indel, corresponding to a compound indel consisting of a 1 bp insertion paired with the 61 bp excision event. DECODR, however, also exhibits the ability to determine *which* base is inserted at the cut site, indicating that an adenine was inserted. ICE, while accurate in determining the contributions of each constituent subsequence, is not designed with the ability to infer inserted bases without a provided HDR template to inform the edit proposals. Supplementary Figure S1D–F contains further examples of bulk Sanger sequencing data analyzed by DECODR, TIDE, and ICE.

### DECODR is capable of deconvoluting compound indels

**FIG. 4.**
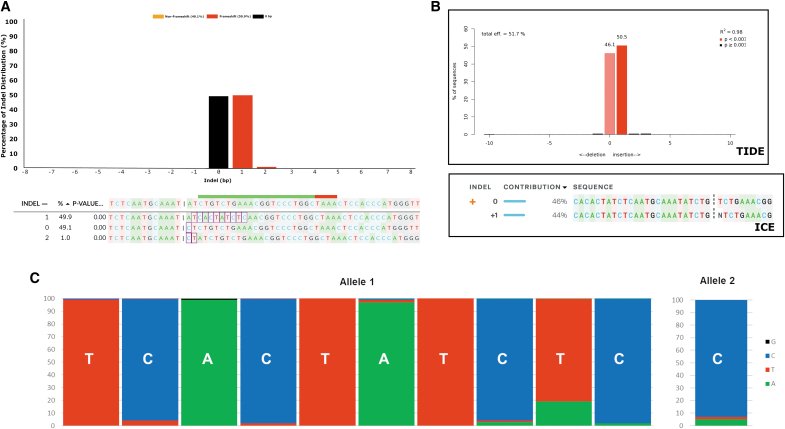
DECODR can recognize and deconvolute compound indels. A single-guide knockout was performed on Hel92.1.7 cells, targeting the two-copy *HBG1* gene. **(A)** Using DECODR, a net +1 bp indel shown in the sequence pane is shown to actually be a 9 bp deletion paired with a 10 bp novel insertion. The other allele contained a single 1 bp change at the cut site (a deletion of 1 bp and an insertion of 1 bp). A third sequence comprising 1% of the overall convoluted sequence was also output by the algorithm, which is likely due to background in the experimental sequence. **(B)** Using both TIDE and ICE, this genetic change is not seen; TIDE shows the net indel size, which is inaccurate to the scope of the genetic change, and ICE reports the sequence is a mixture of wild type and a 1 bp insertion. **(C)** Each inserted base was determined using DECODR's inserted base determination algorithm. The probabilities of each inserted base are shown for each major allele, with the most likely base labeled in white. These per-position basecall determinations are displayed in the DECODR output.

Compound indels are difficult to analyze for functional change of the gene using the edit proposition approach. In large compound indels, a significant number of bases can be deleted, significantly altering gene function. However, because other bases are inserted at the cleaved DNA ends, the overall observed length of the indel is not indicative of the total size of the disruption analyzed. With the exception of HDR-mediated compound indels with a known HDR template to use in edit proposal generation, insertions of greater than a single base cannot be fully deconvoluted using TIDE or ICE; manual deconvolution via allelic analysis was the only way to determine the presence and impact of compound indels accurately, which limited analysis to simple-to-interpret convoluted samples. We tested DECODR's ability to deconvolute these compound indels in a clonal sample isolated while examining Cas12a knockouts in Hel92.1.7 cells (Fig. 4A). The upper panel shows the histogram of detected indels. When compared to the TIDE readout for the same sequence (Fig. 4B, upper panel), a similar net indel distribution can be seen. However, the bottom panel of Figure 4A reveals that the net +1 bp insertion is not a single base inserted, but rather a 10 bp insertion paired with a 9 bp deletion on the same allele. Likewise, the 0 bp sequence is not an unedited wild-type sequence, but rather contains an A > C base change directly at the end of the 5′ overhang of the Cas12a cut site. The ICE readout for the sequence, in the bottom panel of Figure 4B, does not show either of these genetic changes; in line with the TIDE readout, ICE reports one wild-type allele and one allele with a single 1 bp insertion. This analysis is strictly incorrect, as confirmed by manual sequence deconvolution.

## Discussion

DECODR was developed to analyze Sanger sequencing performed on both clonal and bulk populations of cellular DNA after CRISPR cleavage. Due to the endless range and variability of initial conditions utilized in CRISPR gene editing, there was need for an analysis tool that could scale to be able to analyze a wider range of potential indels, as well as one that could be rapidly iterated to keep up with the needs of analysis. Utilizing a novel deconvolution algorithm that smartly generates edit proposals based on initial reading of the edited Sanger sequence, DECODR can accurately determine simple indel events without any limit on range. DECODR can also determine identities of not just deleted bases but inserted bases as well, allowing for a highly accurate readout of Sanger data that can be used to determine better how a CRISPR experiment impacts a targeted gene of interest, at both a bulk and a clonal level.

Though a majority of indel formation in many designed CRISPR experiments may fall within a short range of small indels, larger deletions are more likely to disrupt a target gene. Gene-editing experiments that generate larger deletions are therefore more likely to contain suitable disruptions for any given gene of choice. The designs of current deconvolution tools, however, are not able to handle large deletions; the presence of indels larger than the capacity of previous deconvolution tools can even prevent the accurate determination of shorter indels within the same sequence. In Figure 2B, the K562 clone GATA1KO-117 will not display an accurate contribution of the single T insertion using any previously available deconvolution tools, as the presence of the −115 + 1 bp indel prevents the accurate determination of any contributing sequences (Supplementary Fig. S2). Though both TIDE and ICE both display a +1 bp insertion as their highest percentage contribution, the artificially high background caused by an inability to identify a large deletion properly makes these tools unsuitable to identify the most appealing knockout candidates accurately and quickly when challenged with samples containing such indels. In addition, further experimentation with the *in vitro* gene editing system yielded accurate contributions and indel identities at both clonal and bulk levels, regardless of indel size (Supplementary Fig. S3).^26–28^ Comparisons of Sanger data analyzed via DECODR were also comparable to NGS data analyzed using CRIS.py (Supplementary Fig. S4).^21^

While the increase in measurable indel range offered by DECODR is undoubtedly useful in many use cases, DECODR is still limited by the quality of the input sequences. Factors such as misaligned subpeaks and decreased sequence quality at longer reads can still lead to misattributed basecalls and indel lengths. Indeed, the increased resolution that DECODR offers in terms of inserted base identity causes DECODR to be even more sensitive to these quality issues. In cases where the DNA cleavage sites are far downstream within the analyzed sequence (examples used for testing include but are not limited to 700 bp Sanger sequencing files with the cleavage site at position 550), the decreased peak height and sequence quality around the CRISPR cleavage site will commonly lead to low percentage compound indels being displayed at percent contributions as high as 8% in clonal populations (data not shown). For this reason, it is highly recommended that when sequencing for analysis with DECODR, the cleavage site is located between positions 100 and 300 of the analyzed sequence if possible. In cases where such parameters are not possible, using DECODR to analyze both forward and reverse sequencing can also help cull artifacts.

Compound indels are not common in CRISPR-Cas9-mediated gene editing, as NHEJ as a DNA repair mechanism is more highly weighted with blunt-end DSBs.^8^ Cas9 also recognizes a protospacer adjacent motif (PAM) cleavage site, meaning that indels heavily curtail further cleavage of the DNA, leading to smaller overall indel sizes.^29,30^ In the presence of the PAM-distal staggered ends caused by CRISPR-Cas12a cleavage, however, larger indels caused by MMEJ are much more common.^31,32^ These MMEJ-mediated indels are more complex and more readily form compound indels. These compound indels are not able to be suitably analyzed using either TIDE or ICE. Utilizing DECODR, however, these compound indels can be fully analyzed and incorporated into potential editing outcomes.

The presence of misaligned, off-center sub-sequences can cause incorrect analyses with all currently available sequence deconvolution tools, including TIDE and ICE. DECODR's sub-sequence base-calling algorithm utilizes local maximum subpeak intensities, making these contribution determinations generally more accurate. However, heavily misaligned sub-sequences can still lead to a misinterpretation of total indel length. Sub-sequence misalignment tends to be progressive (i.e., sub-sequences offset by 10% of the main peak height that will have their net indel size increased by 1 bp are measured 10 bp away from the cut site, 2 bp if measured 20 bp away from the cut site, etc.). The larger range of indels and higher resolution on sequence decomposition offered by DECODR can lead to incorrect analyses of these sequences, especially within bulk sequences, where such perturbations in sequence quality may not be as readily apparent on the chromatogram.

The kinetics of PCR reactions may cause shorter amplicons in a mixed-length population to be preferentially amplified over longer amplicon fragments.^33,34^ This might skew the overall ratio of template DNA sub-sequences that is input into Sanger sequencing and lead to an inflation of the contribution of whichever subsequent fragment has the largest deletion. This relationship is not linear, however, and research is underway in our laboratories to determine an implementable formula to correct for this. Because of the capability of DECODR to recognize a larger range of indels, DECODR is potentially more sensitive to this contribution disparity than other size-limited deconvolution tools.

Moving forward with further developing the deconvolution algorithm utilized in DECODR may increase the accuracy and widen the use cases for the software. One potential avenue to do this would be to explore the utilization of machine learning in order to augment or even replace the linear regression model for deconvolution.

## Conclusion

DECODR can be scaled to determine any indel of any size, allowing for future implementation of the DECODR software in targeted endonuclease-based gene-editing strategy, regardless of complexity or scope of edit. The accurate identification and output of compound indels allows for DECODR analysis to be executed in experiments utilizing potentially any configuration of donor DNA sequences, CRISPR-Cas, and endogenous DNA repair pathways.

## Supplementary Material

Supplemental data

Supplemental data

Supplemental data

Supplemental data

Supplemental data

Supplemental data
