# Supplementary material for: Deconvolution of Complex DNA Repair (DECODR): Establishing a Novel Deconvolution Algorithm for Comprehensive Analysis of CRISPR-Edited Sanger Sequencing Data
Source: CRISPR J. 2021 Feb 19;4(1):120–31. doi: 10.1089/crispr.2020.0022 (PMC7898406; doi:10.1089/crispr.2020.0022)
Supplement: Supplemental data [file Supp_TableS1.pdf]

Supplemental Table S1: Polymerases and Primers used for PCR amplification of all utilized gene products

Gene Target	Polymerase	FWD Primer (5' > 3')	REV Primer (5' > 3')
GATA1	Amplitaq	GGGAGGTGGGAAGGAGAAATATGGAG	CCTCACAGTGGTATTCTGACCTAGCC
TRM10C	Phusion	CATGGCTTACGAAAATTATATG	GTATCATTTGATCTAAGGTGAG
NRF2	Q5	ATTAAACAAGGGTGGGATTTCTTCTC	AACTCAGGTTAGGTACTGAACTCATCA
Rb1	Phusion	CAAAGTCACTGGCGTTGAATTG	GTGGTAGGATTACAGGCATGAAC
HBG1	Amplitaq	CCTTAGAAACCACTGCTAACT	GATAGTAGCCTTGTCCTCCTCTG
