# Supplementary material for: Deconvolution of Complex DNA Repair (DECODR): Establishing a Novel Deconvolution Algorithm for Comprehensive Analysis of CRISPR-Edited Sanger Sequencing Data
Source: CRISPR J. 2021 Feb 19;4(1):120–31. doi: 10.1089/crispr.2020.0022 (PMC7898406; doi:10.1089/crispr.2020.0022)
Supplement: Supplemental data [file Supp_TableS2.pdf]

Supplemental Table S2: Open Source Software and Libraries used in the development of DECODR

Front-End UI Module		Back-End Computational Module	
**React.js**	https://reactjs.org	**Django**	https://www.djangoproject.com
**Material UI**	https://material-ui.com	**Django-cors-headers**	https://pypi.org/project/django-cors-headers/
**Axios**	https://www.npmjs.com/package/axios	**Django REST Framework**	https://www.django-rest-framework.org
**Bootstrap**	https://getbootstrap.com	**Celery**	http://www.celeryproject.org
**Chart.js**	https://www.chartjs.org	**Redis**	https://redis.io/
**Rc-slider**	https://www.npmjs.com/package/rc-slider	**XlsxWriter**	https://xlsxwriter.readthedocs.io/
**React-dropzone**	https://www.npmjs.com/package/react-dropzone	**jsonpickle**	https://jsonpickle.github.io/
**Nanobar**	http://nanobar.jacoborus.codes/	**BioPython**	https://biopython.org
**ReactN**	https://www.npmjs.com/package/reactn	**NumPy**	https://numpy.org
**React Sticky Table**	https://www.npmjs.com/package/react-sticky-table	**SciPy**	https://www.scipy.org
**React Select**	https://react-select.com/home	**Matplotlib**	https://matplotlib.org
**React-modal**	https://www.npmjs.com/package/react-modal	**Scikit-learn**	https://scikit-learn.org/stable/
**Reactour**	https://github.com/elrumordelaluz/reactour/issues/68		
