# Supplementary material for: Deconvolution of Complex DNA Repair (DECODR): Establishing a Novel Deconvolution Algorithm for Comprehensive Analysis of CRISPR-Edited Sanger Sequencing Data
Source: CRISPR J. 2021 Feb 19;4(1):120–31. doi: 10.1089/crispr.2020.0022 (PMC7898406; doi:10.1089/crispr.2020.0022)
Supplement: Supplemental data [file Supp_FigS1.pdf]

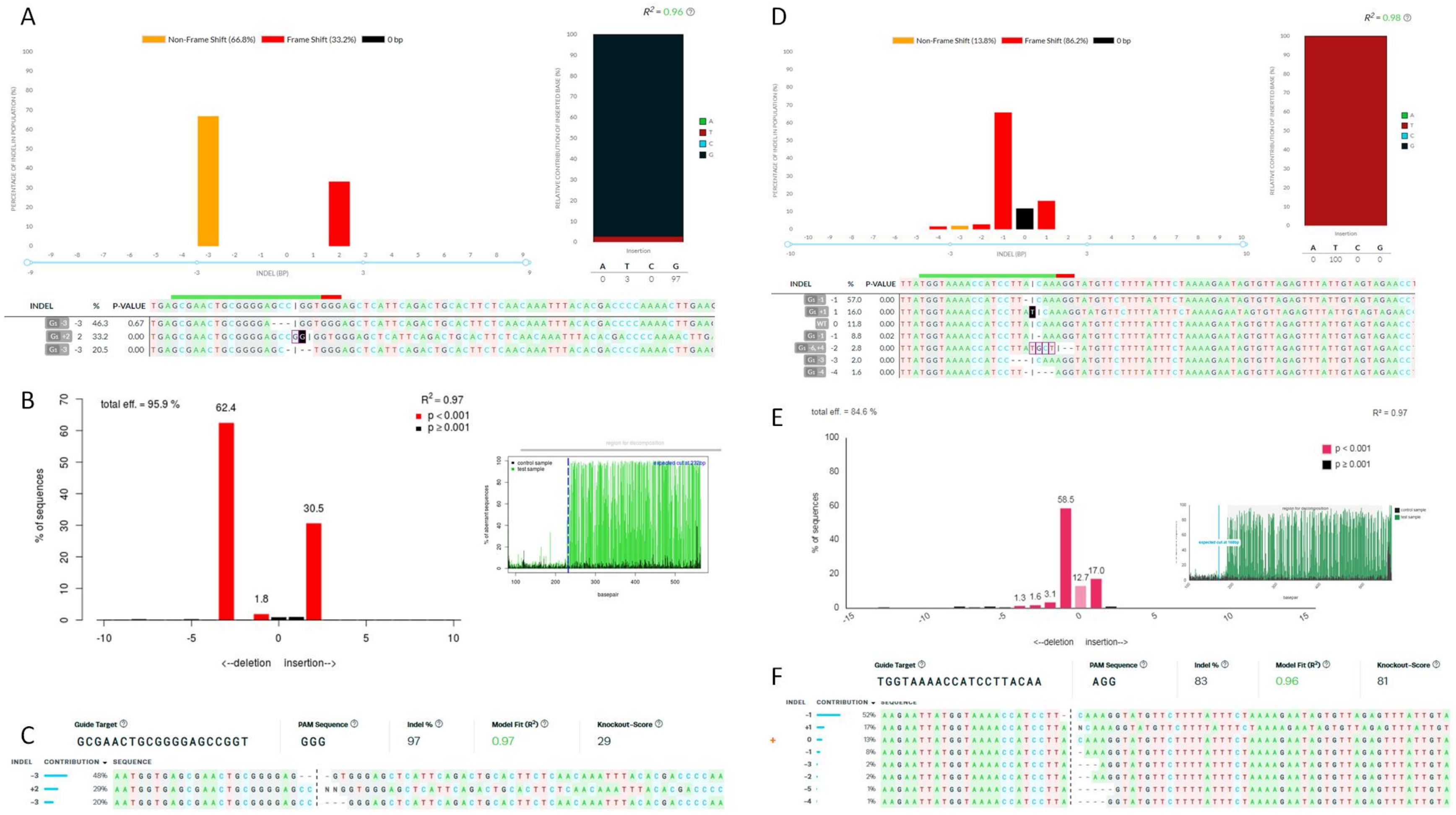

Supplemental Figure S1: Further comparisons of editing efficiency analysis between DECODR, TIDE and ICE. (A-C) Hs766t cells were edited with a single gRNA targeting the PARG gene and clonally expanded. One sequence of one clone with three alleles is displayed here, analyzed with (A) DECODR, (B) TIDE and (C) ICE. All three methods determine the same indel sizes, with similar distributions. Using DECODR, it can be determined that the inserted bases in the recognized +2 insert are two guanines. TIDE uniquely lists a statistically significant indel at -1, making up only 1.8 percent of the total sequence. Due to the clonal nature of this analysis, this value, though showing a p-value <0.01, was dismissed as a sequencing artifact due to low contribution. (D-F) NCI-H226 cells were edited with a single gRNA targeting the TACE gene, and analyzed as a

bulk population to determine knockout efficiency. The bulk population was analyzed via (D) DECODR, (E) TIDE and (F) ICE. All three methods list determine similar indel distributions among the bulk populations. Using DECODR, it can be determined that the inserted base directly at the cleavage site is an inserted thymine. DECODR also determines the presence of a compound indel making up 2.8 percent of the total sequence, where 6 bases were deleted and 4 bases were inserted. Due to low overall contribution, further experimentation would need to be performed to validate the presence of this sequence within the population. Unlike the clonally expanded population, however, none of the determined sequences can be disregarded due to low overall contribution.
