# Supplementary material for: Deconvolution of Complex DNA Repair (DECODR): Establishing a Novel Deconvolution Algorithm for Comprehensive Analysis of CRISPR-Edited Sanger Sequencing Data
Source: CRISPR J. 2021 Feb 19;4(1):120–31. doi: 10.1089/crispr.2020.0022 (PMC7898406; doi:10.1089/crispr.2020.0022)
Supplement: Supplemental data [file Supp_FigS2.pdf]

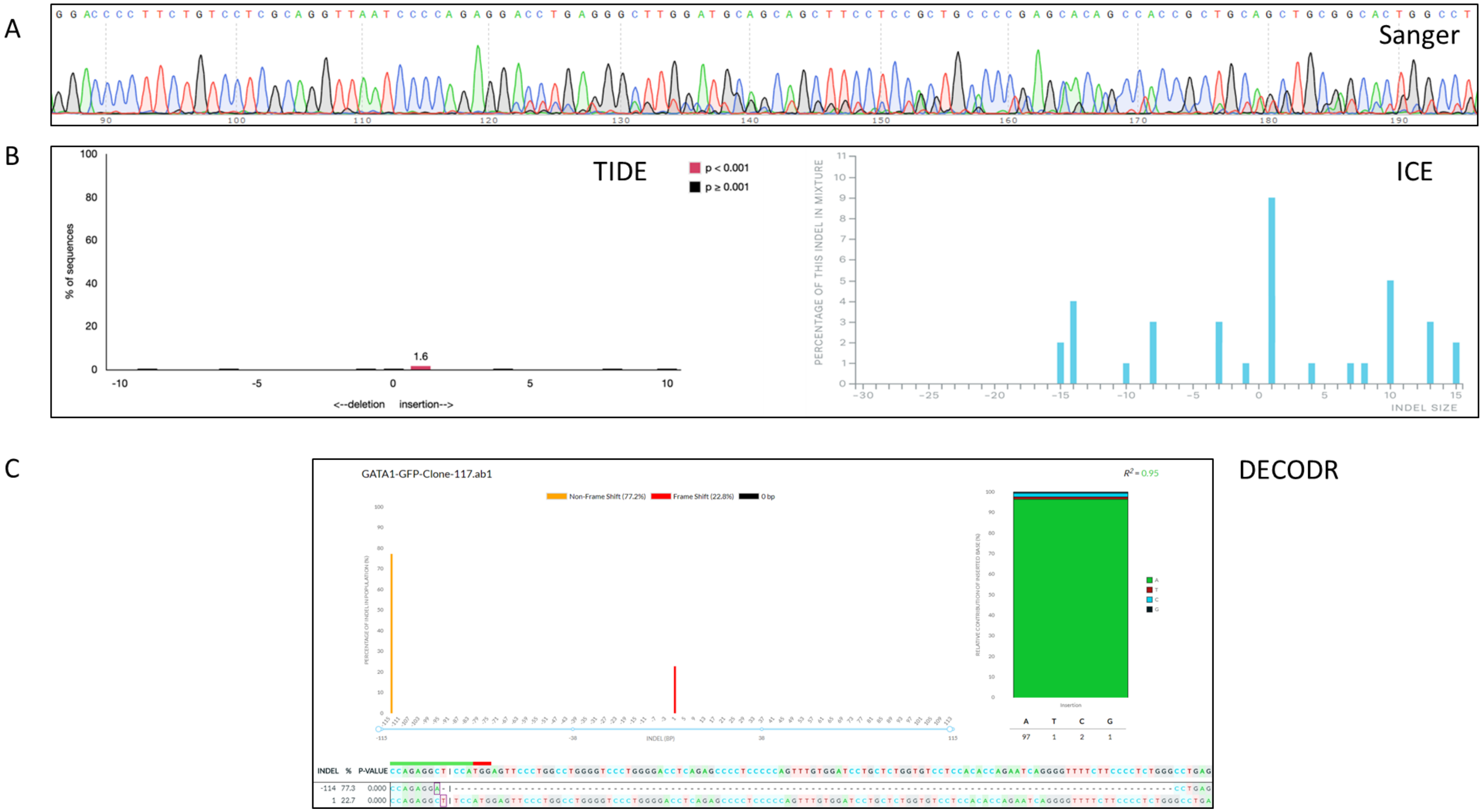

Supplemental Figure S2: Comparison of TIDE, ICE and DECODR outputs of large and compound indel sequences. (A) Sanger sequencing output of GATA1 clone detailed in Figure 3B, containing a (-115, +1)bp indel in one allele and a +1bp indel in the other. (B) TIDE and ICE analysis outputs for the GATA1 clone. (C) Complete DECODR output for the GATA1 clone, displaying indel histogram, deconvoluted sequences, and the base insertion probability for the single A insertion on the +1bp allele.
