# Supplementary material for: Deconvolution of Complex DNA Repair (DECODR): Establishing a Novel Deconvolution Algorithm for Comprehensive Analysis of CRISPR-Edited Sanger Sequencing Data
Source: CRISPR J. 2021 Feb 19;4(1):120–31. doi: 10.1089/crispr.2020.0022 (PMC7898406; doi:10.1089/crispr.2020.0022)
Supplement: Supplemental data [file Supp_FigS3.pdf]

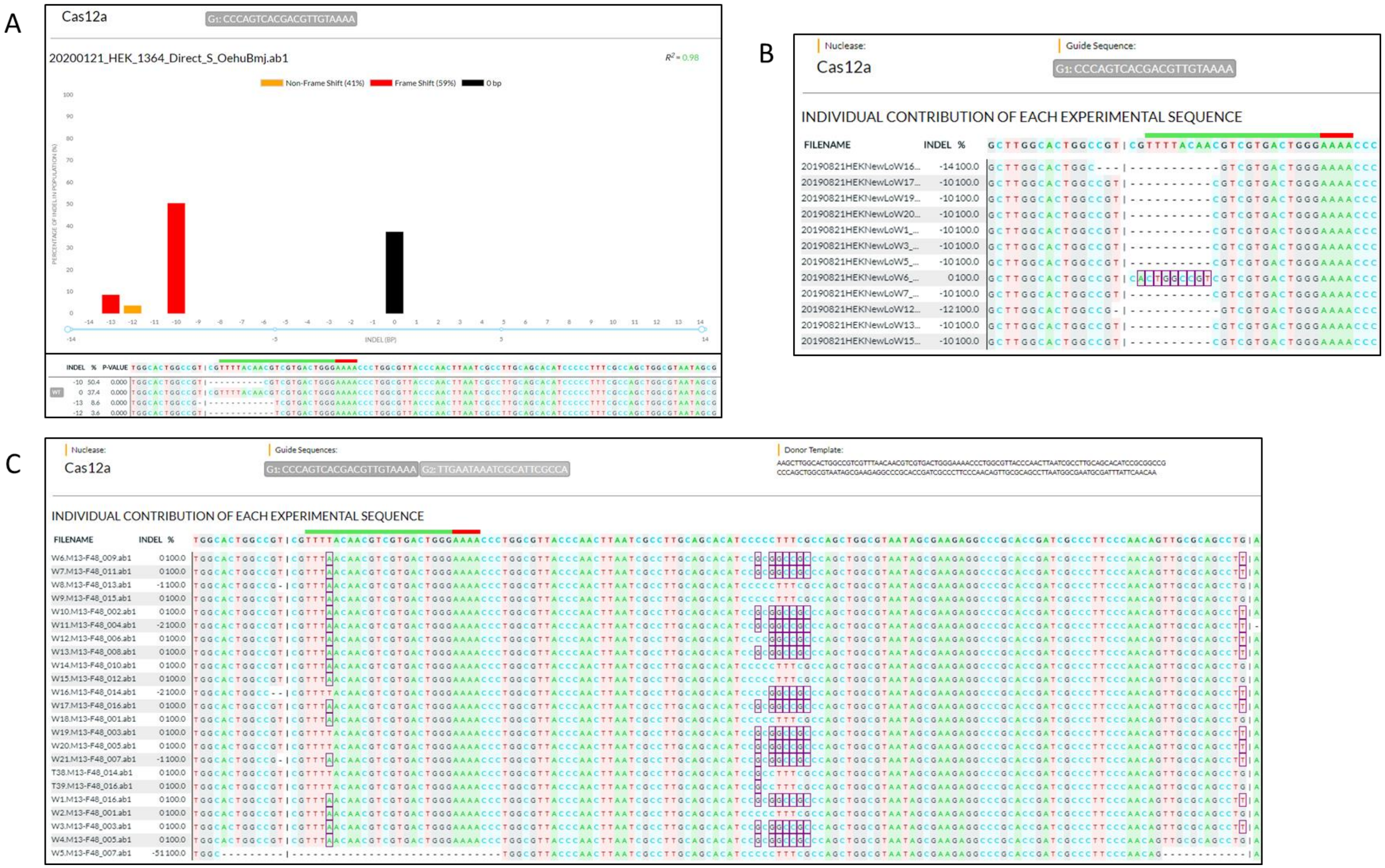

Supplemental Figure S3: DECODR is efficient at analyzing multiple edited DNA files simultaneously within an *in vitro* testbed. (A) Single-allele pure sequences were generated using the *in vitro* gene editing system using a single Cas12a guide sequence and no donor template. Sequences were then analyzed via DECODR in a single batch run. (B) Single-allele pure sequences were generated via the *in vitro* system utilizing two guide sequences and a homology donor containing 5’ and 3’ single-base barcodes and a diagnostic sequence change in the middle.
