# Supplementary material for: Deconvolution of Complex DNA Repair (DECODR): Establishing a Novel Deconvolution Algorithm for Comprehensive Analysis of CRISPR-Edited Sanger Sequencing Data
Source: CRISPR J. 2021 Feb 19;4(1):120–31. doi: 10.1089/crispr.2020.0022 (PMC7898406; doi:10.1089/crispr.2020.0022)
Supplement: Supplemental data [file Supp_FigS4.pdf]

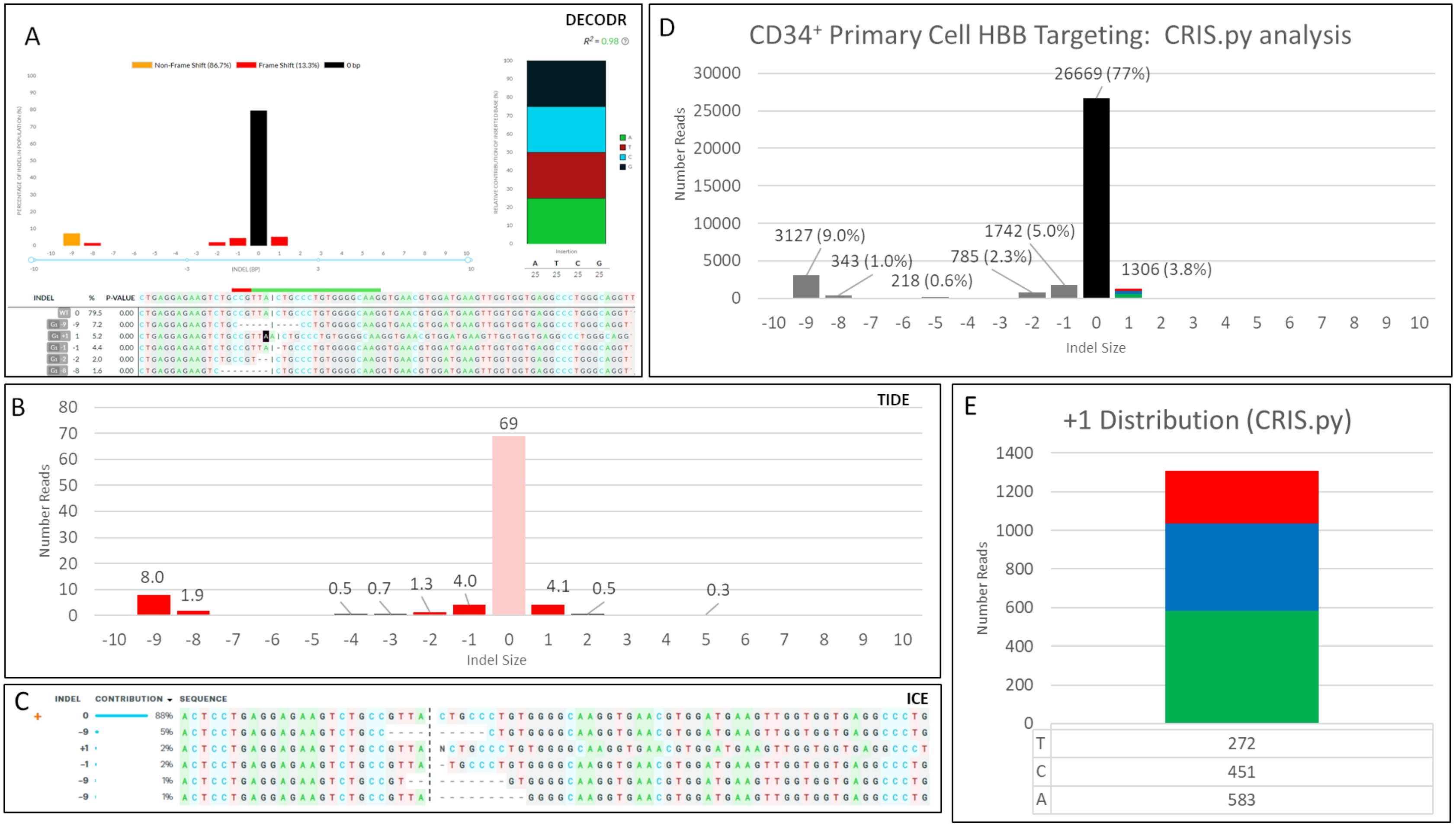

Supplemental Figure S4: Comparison of data analysis quality between DECODR and Next Generation Sequencing in bulk sequenced populations. For these data, bulk DNA was isolated from a population of CD34 cells edited with a HBB-targeting CRISPR/Cas9 complex. This DNA was utilized to generate both Sanger sequencing data and Next-Generation Sequencing data. (A-C) display the editing outcomes depicted by analyzing the Sanger sequencing data with (A) DECODR, (B) TIDE, and (C) ICE. (D) depicts analysis of the NGS Data that was gathered utilizing the CRIS.py analysis tool (available at https://github.com/patrickc01/CRIS.py). The +1 insertion which made up 3.8% of the total analyzed reads consisted of three different +1 insertions at the same position. (E) shows the distribution of this +1 insertion, with raw read numbers shown below.
